# Atomistic-Continuum Study of an Ultrafast Melting Process Controlled by a Femtosecond Laser-Pulse Train

**DOI:** 10.3390/ma17010185

**Published:** 2023-12-29

**Authors:** Yu Meng, An Gong, Zhicheng Chen, Qingsong Wang, Jianwu Guo, Zihao Li, Jiafang Li

**Affiliations:** 1 Laser Micro/Nano Fabrication Laboratory, School of Mechanical Engineering, Beijing Institute of Technology, Beijing 100081, China; 2 Yangtze Delta Region Academy of Beijing Institute of Technology, Jiaxing 314019, China; 3 School of Physics, Beijing Institute of Technology, Beijing 100081, China

**Keywords:** femtosecond laser, ultrafast melting process, molecular dynamics, two-temperature model, laser-material interaction

## Abstract

In femtosecond laser fabrication, the laser-pulse train shows great promise in improving processing efficiency, quality, and precision. This research investigates the influence of pulse number, pulse interval, and pulse energy ratio on the lateral and longitudinal ultrafast melting process using an experiment and the molecular dynamics coupling two-temperature model (MD-TTM model), which incorporates temperature-dependent thermophysical parameters. The comparison of experimental and simulation results under single and double pulses proves the reliability of the MD-TTM model and indicates that as the pulse number increases, the melting threshold at the edge region of the laser spot decreases, resulting in a larger diameter of the melting region in the 2D lateral melting results. Using the same model, the lateral melting results of five pulses are simulated. Moreover, the longitudinal melting results are also predicted, and an increasing pulse number leads to a greater early-stage melting depth in the melting process. In the case of double femtosecond laser pulses, the pulse interval and pulse energy ratio also affect the early-stage melting depth, with the best enhancement observed with a 2 ps interval and a 3:7 energy ratio. However, pulse number, pulse energy ratio, and pulse interval do not affect the final melting depth with the same total energies. The findings mean that the phenomena of melting region can be flexibly manipulated through the laser-pulse train, which is expected to be applied to improve the structural precision and boundary quality.

## 1. Introduction

Femtosecond lasers play an important role in micro- and nano-fabrication because of the advantages of high energy intensity and small collateral damage [1,2,3,4,5]. The interaction between femtosecond lasers and metallic materials can be divided into two stages [1,6,7]. Firstly, upon femtosecond laser irradiation, the electron sub-system absorbs laser energy as a result of a sharp electron temperature increment. The lattice sub-system remains at a low temperature, resulting in thermal nonequilibrium between the electron sub-system and the lattice sub-system [8,9,10,11]. Secondly, this nonequilibrium drives electron-phonon coupling energy transport. The whole relaxation process between the two sub-systems lasts several picoseconds and is accompanied by the combined effect of electron heat conduction and electron-phonon coupling [8,9]. Eventually, the increment of lattice temperature triggers photo-mechanical and photo-thermal effects, leading to an abrupt phase change [10,12,13,14].

By conducting further research on such nonequilibrium and nonlinear processes, it is possible to exert control over the corresponding phase-change phenomena to meet diverse processing requirements, these include reducing the heat-affected zone (HAZ) [1,2,7,12,15], minimizing the generation of microcracks [12,15,16] and recasts [15,17], facilitating nanoparticle sintering [5,15,18,19,20], and achieving highly localized material removal [12,21,22].

In recent decades, a novel melting phenomenon known as homogeneous melting has been discovered. Distinguished from the conventional melting process (heterogeneous melting [23]), when subjected to femtosecond laser irradiation, the metal undergoes rapid heating and reaches a highly superheated state. Subsequently, the liquid nucleation process takes place homogeneously, leading to complete melting within a few picoseconds [23,24].

Its mechanism has prompted extensive theoretical, experimental, and simulation investigations [4,8,24,25,26,27]. The transition from the solid phase to the liquid phase of Al was observed via ultrafast electron diffraction (UED) by Siwick et al. [25]. Rethfeld et al. proposed the theory of ultrafast thermal melting in laser-excited solids, specifically referring to the phenomenon of homogeneous nucleation [24]. The formation mechanism of the two melting phenomena was further analyzed by Ivanov and Zhigilei using the molecular dynamics coupling two-temperature model (MD-TTM model) [8]. The transition from heterogeneous to homogeneous melting at higher energy densities is visualized through UED by Mo et al. [4]. The influence of electron heat conduction and electron-phonon coupling on laser-metal interaction was elucidated by Ivanov et al., and the pulse duration and the laser fluence could affect the above processes [10]. In general, under femtosecond laser irradiation, the metals are heated to a sufficiently high temperature where heterogeneous nucleation is suppressed and homogeneous nucleation predominates. Consequently, the system undergoes catastrophic homogeneous nucleation and completes the phase change within a few picoseconds.

The aforementioned research demonstrates that the control of ultrafast melting processes encounters significant challenges attributed to the imperative requirement for achieving electron dynamic regulation. Fortunately, the electron dynamics control (EDC), by the shaping femtosecond laser-pulse train, can realize the effective control of femtosecond laser fabrication at the electron level [1]. Significant advancements have been made by precisely designing laser-pulse trains to modify thermophysical properties and subsequently alter the phase-change process [1,28]. Li et al. discovered that the pulse train can alter energy transport and associated phase change, enabling control over the size distribution of metal nanoparticles [15]. Povarnitsyn et al. unveiled two mechanisms for suppressing ablation irradiated by a double-pulses laser (DP) [29]. Förster and Lewis conducted a comprehensive analysis of the mechanisms active during the different stages of the ablation process induced by a DP [13]. Englert et al. controlled the ionization processes in high-band-gap materials via temporal asymmetric pulse shapes on intrinsic time and intensity scales [30]. The incubation phenomenon during laser ablation with bursts of femtosecond pulses was reported by Gsudiuso et al. [31]. Rong et al. found the advantages of femtosecond laser-pulse train processing in reducing thermal-mechanical damage compared to single-pulse (SP) processing [17].

Despite the wide range of potential applications for laser-pulse trains, current research on the spatiotemporal shaping femtosecond pulses primarily focuses on the ablation phenomenon. There is limited direct research on controlling the melting process with spatiotemporal-shaping femtosecond pulses, particularly with pulse numbers exceeding two. However, the melting process directly determines the quality, efficiency, and precision of the processing. Roy et al. investigated the influence of Cu nanoparticle size and morphology on sintering temperature and sintering quality. It was found that the sintered product of Cu nanoparticles exhibited outstanding mechanical strength and high electrical conductivity [32]. By employing ultrashort laser pulses in assisted powder-bed fusion, Ullsperger et al. discovered a more uniform melting pool shape, along with refined primary Si and eutectic structure during the powder-bed fusion process [33]. Furthermore, advancements in first-principles research have shown that the material properties affected by electron temperature in the MD-TTM model are not suitable to be expressed by classical constant or linear equations [17,34,35].

In this work, firstly, the laser pulse is divided 1:1 by the combination of beam splitter and mirrors. The response of Al to the time interval and energy at energies slightly below the ablation threshold is investigated by adjusting the time interval and total energy of the double pulses. Secondly, the thermophysical properties of Al obtained by different methods are compared and applied to the MD-TTM model. After the model validation, the significance of temperature-dependent thermophysical parameters in laser-material interactions during the ultrafast melting process is elucidated through comparative analysis. Subsequently, a simplified method, similar to that used in [22,36], was employed to combine the 1D results to obtain 2D results for both the SP and DP with a 2 ps interval, thereby replicating the observed diameter increase in the melting region in the experimental results and analyzing relevant phenomena. Next, based on the verification of SP and DP simulation, the 2D melting region in Al was predicted under five pulses (FP) by employing the same mode. Furthermore, we also simulated the early-stage melting depth of Al when irradiated by a DP. The influence of different pulse numbers, pulse intervals, and pulse energy ratios on the longitudinal melting (propagation direction) phenomena of Al, including differences in melting depth and melting velocity, was investigated, along with their potential mechanisms.

## 2. Experimental and Modeling

### 2.1. Experimental Setup

**Figure 1:**
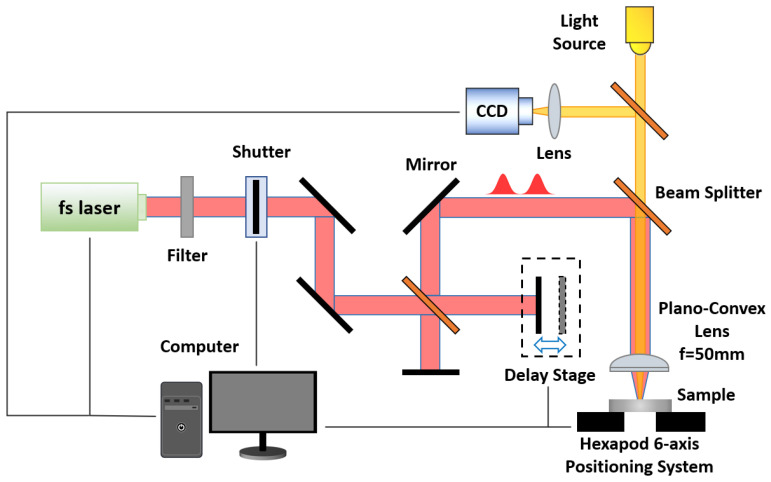
Schematic diagram of the experimental setup of double-pulse laser processing.

Figure 1 illustrates the schematic diagram of the experimental setup. The femtosecond laser source is a Ti: sapphire chirped pulse-amplification (CPA) system (Spectra-Physics Spitfire Ace, Spitfire Pro-35F1KXP, California, CA, USA), operating at a repetition frequency of 1 kHz, with a full width at half maximum (FWHM) of 100 fs and a center wavelength of 800 nm. The laser pulse is split into two equal-energy pulses using a beam splitter (Thorlabs, Inc., Newton, NJ, USA) in a 1:1 split ratio. These two pulses are combined to form a DP train through the application of mirrors and a beam splitter. The time interval between the double pulses is precisely controlled by a one-dimensional translation stage (the minimum linear displacement is 1 μm, Zolix, Inc., Beijing, China). Subsequently, the pulses are focused onto the sample by a plano-convex lens (f=50 mm, Thorlabs, Inc., Newton, NJ, USA). The sample Al (10 mm×10 mm×1.0 mm, one side polished, orient <100>) is positioned on a hexapod six-axis positioning system (M-840.5DG, Physik Instrumente, Inc., Auburn, MA, USA). The microstructures are observed via scanning electron microscopy (SEM, Thermo Scientific Apreo 2C, Thermo Fisher Scientific Inc., Waltham, MA, USA).

To ensure that the experimental results are reliable, we obtained multiple data by keeping the shutter open and moving the sample along a straight line. The average value of these data was then taken as the experimental result. In addition, we imported SEM images into a MATLAB program and uniformly adjusted their contrast ratio. Through this program, we can obtain the SEM images after processing, which have a clear light-dark dividing line. The dividing line is used as the boundary of the melting region. Finally, the length of the major axis and the minor axis is obtained by fitting the elliptic equation according to the dividing line.

### 2.2. The MD-TTM Model

The Al melting process under femtosecond laser-pulse train irradiation is simulated by the MD-TTM model [34,35]. For the atomistic scale, the MD is used to provide a unique atomistic view of the laser-induced phase-change process.

(1) $$m_{i}\frac{\partial v_{i}}{\partial t}=F_{i}\left(t\right)-\gamma_{i}v_{i}+{\tilde{F}}_{ran}\left(t\right)$$

where $m_{i}$ is the mass of atom i with velocity $v_{i}$, $F_{i}$ is the resultant force acting on atom i, which is calculated from the EAM potential [37,38], and $\gamma_{i}$ is the friction term, which directly relates to the electron-phonon coupling factor $G_{e-ph}$, where N is the atom number density, $k_{B}$ is the Boltzmann constant, and ${\tilde{F}}_{ran}$ is the random force from the Langevin dynamics.

(2) $$\gamma_{i}=\frac{m_{i}G_{e-ph}}{3Nk_{B}}$$

(3) $${\tilde{F}}_{ran}(t)=\sqrt{\frac{24k_{B}T_{e}\gamma_{i}}{\Delta t}}{\tilde{v}}_{i}$$

where Δt is the timestep and ${\tilde{v}}_{i}$ is a random vector. The sum of ${-\gamma }_{i}v_{i}$, and ${\tilde{F}}_{ran}$ is in direct proportion to the energy transfer of electron-phonon coupling.

For the continuum scale, the TTM model is used to describe laser energy input, thermal diffusion, and temperature evolution. To establish the coupling between the MD model and the TTM model, the lattice sub-system in the classical TTM model is substituted with the aforementioned MD model.

(4a) $$C_{e}(T_{e})\frac{\partial T_{e}}{\partial t}=\nabla \left[k_{e}(T_{e})\nabla T_{e}\right]-{\left(T_{e}-T_{l}\right)G}_{e-ph}(T_{e},T_{l})+S\left(z,t\right),$$

(4b) $$C_{l}\left(T_{l}\right)\frac{\partial T_{l}}{\partial t}=\nabla \left[k_{l}(T_{l})\nabla T_{l}\right]+{\left(T_{e}-T_{l}\right)G}_{e-ph}(T_{e},T_{l})$$

where C is the volumetric heat capacity, k is the thermal conductivity, and T is temperature. The subscripts e and l, represent electrons and lattices, respectively. $G_{e-ph}$ is the electron-phonon coupling factor. In this work, the thermophysical parameters ($C_{e},k_{e},G_{e-ph}$) of Al are determined by $T_{e}$ and $T_{l}$, which are discussed in Section 3.2. The last term in the right hand of Equation (4a) is the laser source $S\left(z,t\right)$, which is a Gaussian beam that decays along the laser irradiation direction.

(5) $$S\left(z,t\right)=\sum_{i=1}^{N_{laser}}\left[1-R\right]\frac{2}{\sqrt{\pi /ln2}t_{p}}\frac{\eta_{i}F_{ab}}{\left(L_{op}+L_{ball}){(1-e}^{-\frac{z}{L_{op}+L_{ball}}}\right)}e^{-[\left(4ln2\right)\frac{{\left(t-3t_{p}-t_{i}\right)}^{2}}{t_{p}^{2}}+\frac{z}{L_{op}+L_{ball}}]}$$

where i is the pulse number of the current calculation, $N_{laser}$ is the total pulse number, R is the optical reflectivity (0.88 [37]), $\eta_{i}$ is the proportion of the ith pulse to the total energy, $F_{ab}$ is the total absorbed laser fluence, t is the time of the current calculation, $t_{p}$ is the pulse duration, $t_{i}$ is the pulse interval between the *i*th and (*i* − 1)th sub-pulses, $L_{op}$ is the optical penetration depth, which equals the reciprocal of the optical absorption coefficient, $L_{ball}$ is the ballistic transportation length (16 nm), and z is the depth.

It should be emphasized that the above MD-TTM model is only applicable to thermal equilibrium conditions, and the solution of non-thermal equilibrium processes needs to use the hyperbolic two-temperature model (HTTM) proposed by Sobolev [39]. Nevertheless, the duration of the laser (100 fs) in this work is much longer than the relaxation time of aluminum [40], in this case, the HTTM degenerates into the TTM [39]. Hence, it is justifiable to employ MD-TTM for analyzing the ultrafast melting phenomenon.

### 2.3. Simulation Details

#### 2.3.1. Simplified Method and Computational Domain Settings

In the case of ablation simulations, the relatively large [41] lateral size (laser spot radius direction, the top 150 nm surface region consists of 84.2 million atoms) can present more abundant phenomena, such as surface melting, rapid resolidification, and generation of voids and a nanocrystalline layer in the sub-surface region [41,42], while smaller sizes cannot. It is well known that atomistic dimensions are in the length scale of Angstrom (Å) [25]. Therefore, simulations to match the actual manufactured size in micrometers would require tens to millions of atoms, which requires huge computational resources. Therefore, an appropriate simplification method is a premise used to achieve the research goal of this work. Fortunately, this problem can be solved by the simplified method proposed by Kumar et al. [36] and applied to the MD-TTM model by Zhou et al. [22].

**Figure 2:**
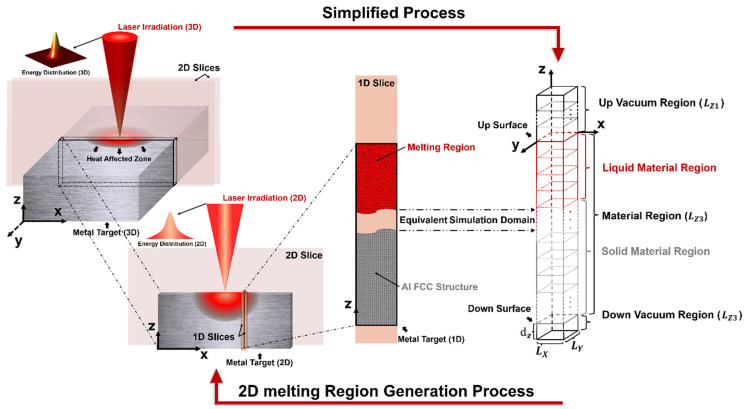
Simplified dimensional schematic diagram of the Al melting process under laser irradiation and the design of 1D equivalent computational domain.

Figure 2 reveals the whole process by using this method to simplify the 3D manufacturing problem into a 1D computational domain. First of all, the laser energy and melting region have a full-scale axisymmetric feature. A 2D computational domain is directly simplified from the 3D computational domain. Next, the 2D computational domain is divided into a series of 1D computational domains. The position of the 1D computational domain in the 2D computational domain determines the total energy it can obtain. It must be noted that this division will indeed affect the calculations of lateral and longitudinal heat transfer, but at the nanoscale subdivision setting, the temperature difference between the 1D computational domain and the 2D computational domain is less than 2% in Zhou et al.’s work [22].

For the 1D computational domain, a similar method was used in our previous work [43]. The laser irradiated on the Al surface along the negative z-direction. Two vacuum regions [27,43] were added to the up surface and down surface of the material region to ensure that atoms did not escape from the computational domain due to material expansion. The whole system was divided into grids with independent $T_{e}$ and $T_{l}$. The temperature of the material was initially set to room temperature: 298.15 K. The size of the differential grid was 4.05 nm×4.05 nm×1.0125 nm. Furthermore, according to the findings in [11,44,45], it has been observed that when the film thickness exceeds 100 nm, any further increase in thickness has a negligible impact on the surface laser-material interaction. This finding enabled us to accurately replicate the experimental results obtained from bulk samples (10 mm) through the simulation of 113.4 nm. The lattice sub-system was created using the classical MD method and equilibrated at 298.15 K for 50 ps [43] in terms of the canonical ensemble (NVT) to complete the initialization and relaxation, which are seen in Figure 2. The grids are colored in red and grey to represent the liquid region and solid region, respectively. The colors also agree with the colors in the atomistic snapshots in Section 3. For the convenience of readers, all the parameters in this work are listed in the Nomenclature.

#### 2.3.2. Computational Flowchart

**Figure 3:**
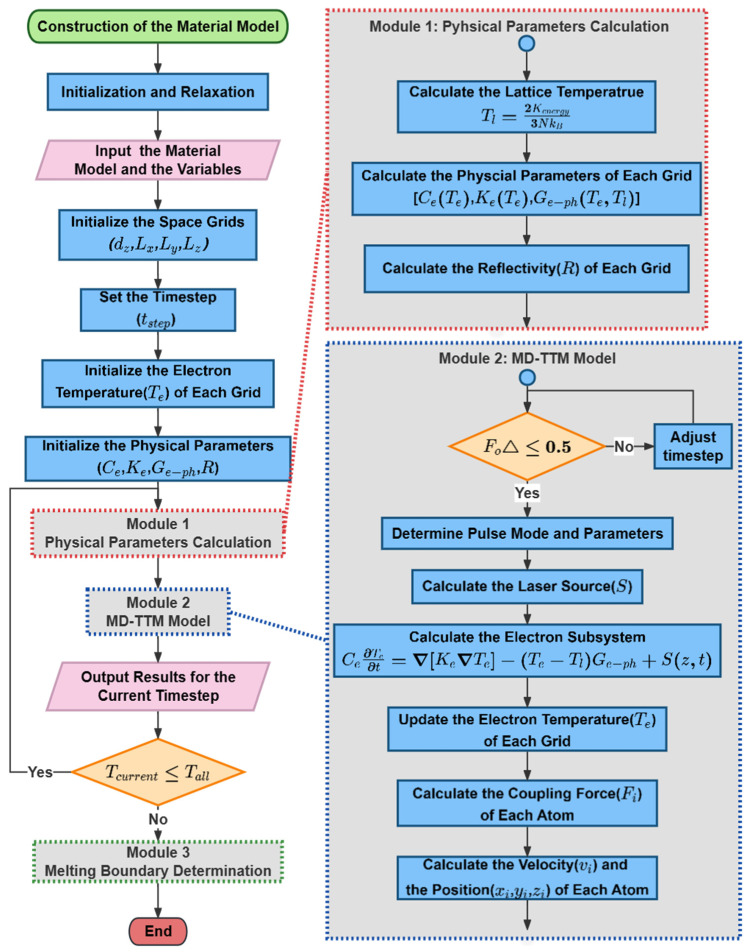
Flowchart of the MD-TTM model incorporating dynamic thermophysical parameters.

Benefiting from previous works [34,35,46,47,48], the classical MD-TTM model has already been integrated into the Large-scale Atomic/Molecular Massively Parallel Simulator (LAMMPS) [49]. On this basis, two new modules were developed and integrated into the LAMMPS program. The details are illustrated in Figure 3. At the beginning of each loop, the program first runs the developed Module 1 named Physical Parameters Calculation. The kinetic energy of atoms was collected to calculate the $T_{l}$ of each grid through Equation (6) [49]

(6) $$T_{l}=\frac{2K_{e}}{3Nk_{B}}$$

where $K_{e}=\sum_{i=1}^{N}\frac{1}{2}m_{i}v_{i}^{2}$ is the total kinetic energy of atoms in the grid. Secondly, the new thermophysical parameters of each grid are updated by solving Equations (8)–(10). Eventually, all the thermophysical and optical parameters of Al involved in the MD-TTM model were updated and input into Module 2 (the MD-TTM model) to complete the whole loop. It should be emphasized that the electron temperature and lattice temperature shown in this work are obtained by simulation.

Moreover, because the finite difference method (FDM) was utilized to solve the TTM model, it is necessary to meet the stability criterion.

(7) $$F_{o_{\Delta }}=\frac{\alpha \Delta t}{\Delta x^{2}+\Delta y^{2}+\Delta z^{2}}\leq 0.5$$

where $F_{o_{\Delta }}$ is the Fourier number, $\alpha =\frac{k_{e}}{C_{e}}$ is the thermal diffusivity, and Δt and Δx, Δy, Δz represent timestep and spacesteps, respectively. If the conditions are not satisfied, the program will report an error.

#### 2.3.3. Laser-Pulse Train

**Figure 4:**
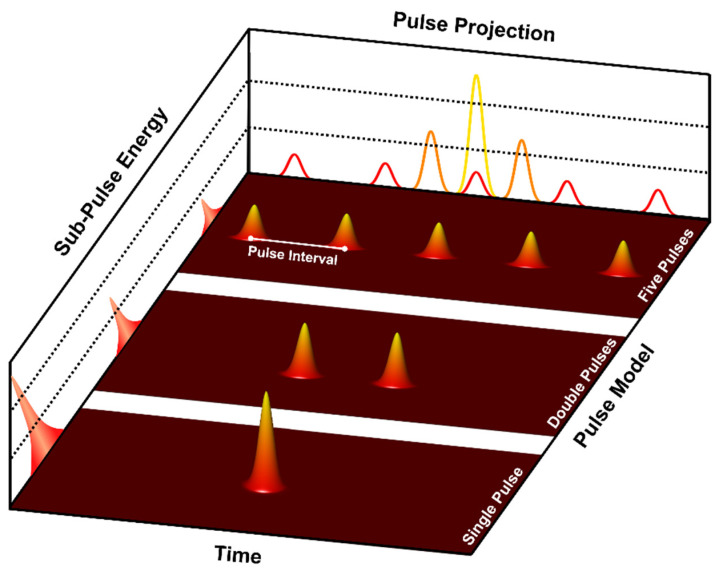
Schematic diagram of the laser-pulse train.

As shown in Figure 4, The spatiotemporal energy profiles throughout the overall femtosecond laser irradiation process are influenced by various factors, such as the pulse number, pulse interval, and energy ratio. When the simulation reaches the “Determine Pulse Mode and Parameters” process of “Module 2” in Figure 3, it will read the laser parameters and generate the desired spatiotemporal shaping laser-pulse train.

#### 2.3.4. Judgement of Energy Conservation

In addition to the stability criterion, to meet the conservation of energy is essential. A series of additional 1D calculations with different aluminum film thicknesses and pulse settings are implemented. Table 1 shows the calculated results of $T_{l}$. It should be noted that to facilitate the comparison, all temperatures are the overall average temperature at 50 ps. As seen from Table 1, it can be found that the temperature difference of multiple pulse modes is within 20 K for the cases with the same energy and thickness. Even for the 500 nm results with smaller $T_{l}$, the error is within 2.5%. This is also shown in the standard deviation σ results. Therefore, the influence of the above errors on the simulation can be ignored.

## 3. Results and Discussion

Firstly, the response of Al to the time intervals and energies strictly below the ablation threshold is explored by adjusting the time interval and the total energy of the double pulses (Section 3.1). Secondly, multiple sets of thermophysical parameters obtained from different experiments and simulations are collected and compared. The impact of the laser-pulse number on the early stage of the melting process (first 50 ps) is conclusively demonstrated by the MD-TTM model incorporating temperature-dependent thermophysical parameters (Section 3.2). Thirdly, the 1D simulation results are assembled to obtain 2D results for an SP and a DP with a 2 ps interval by a simplified method proposed by Kumar et al., enabling an analysis of the lateral differences in the melting phenomenon [36]. Additionally, the same method is employed to predict the results for FP (Section 3.3). Finally, the investigation explores the impact of pulse interval and pulse energy ratio on the longitudinal melting phenomenon in Al by the same model (Section 3.4).

### 3.1. Experimental Results of Al Response to Pulse Interval and Energy

The sample Al is irradiated by an SP and DPs with 1 ps, 2 ps, and 3 ps intervals, respectively, and varying energies. In the case of the DP, the initial laser-pulse energy is divided into two pulses of equal energy.

**Figure 5:**
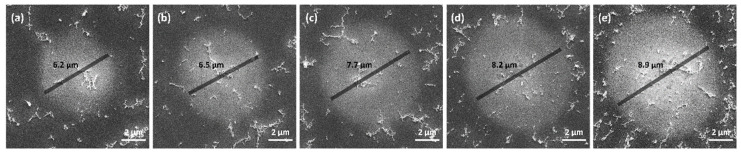
SEM images of melting region for SPs at fluences (**a**) $0.44\;J/{cm}^{2}$, (**b**) $0.50\;J/{cm}^{2}$, (**c**) $0.56\;J/{cm}^{2}$, (**d**) $0.63\;J/{cm}^{2}$, and (**e**) $0.67\;J/{cm}^{2}$.

Figure 5 illustrates SEM images of Al irradiated by SPs with varying energies. The size of the melting region increases as the pulse energy increases.

**Figure 6:**
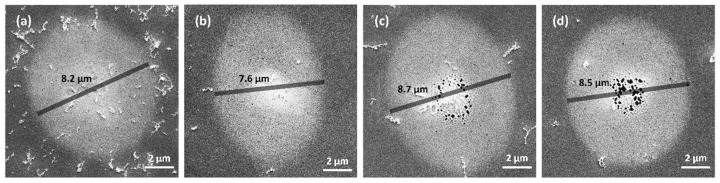
SEM images of melting region for (**a**) an SP and DPs with pulse intervals of (**b**) 1 ps, (**c**) 2 ps, and (**d**) 3 ps, at a total fluence of $0.63\;J/{cm}^{2}$.

Figure 6 shows the SEM images of the melting region of four settings of an SP and DPs with 1 ps, 2 ps, and 3 ps intervals at a total fluence of $0.63\;J/{cm}^{2}$. In terms of the size of the melting region, both DPs with 2 ps and 3 ps intervals demonstrate larger melting compared to the SP (SP: 8.2 μm, the DP with a 2 ps interval: 8.7 μm, the DP with a 3 ps interval: 8.5 μm,). Interestingly, the melting region diameter is the smallest for the DP with a 1 ps interval (7.6 μm). This phenomenon may be attributed to electron excitation, as reported in [50,51,52]. Although these factors may result in reduced energy absorption and subsequently a smaller melting diameter, SEM results for the SP and DPs with 2 ps and 3 ps intervals confirm that multiple pulses enhance melting diameter, even when employing the same or lower laser energy. In addition, when comparing Figure 6c,d, the melting region is smaller for a DP with a 3 ps interval than for a DP with a 2 ps interval.

In terms of the morphology of the melting region, the melting regions for an SP and a DP with a 1 ps interval exhibit high surface smoothness. Voids appear near the center of the melting region for DPs with 2 ps and 3 ps intervals. Specifically, the DP with a 3 ps interval shows more concentrated voids. In addition, in multiple pulse experiments, the areas of melting exhibit an elliptical shape, with the long axis corresponding to the polarization direction. Moreover, as the number of pulses increases, the long axis becomes relatively longer compared to the short axis. This phenomenon is widely seen in other multi-pulse studies [50,53], and to avoid the influence of polarization, we choose the short axis as the melting diameter.

In conclusion, the interval of the DPs affects the diameters and the surface morphology of the melting region. The diameter of the melting region generated by a DP with a 2 ps interval is the biggest with the largest void region. This will be discussed in detail in Section 3.3 and Section 3.4.

### 3.2. Comparison of the Temperature-Dependent Thermophysical Parameters and the Effect of Pulse Number on Simulation Results

**Figure 7:**
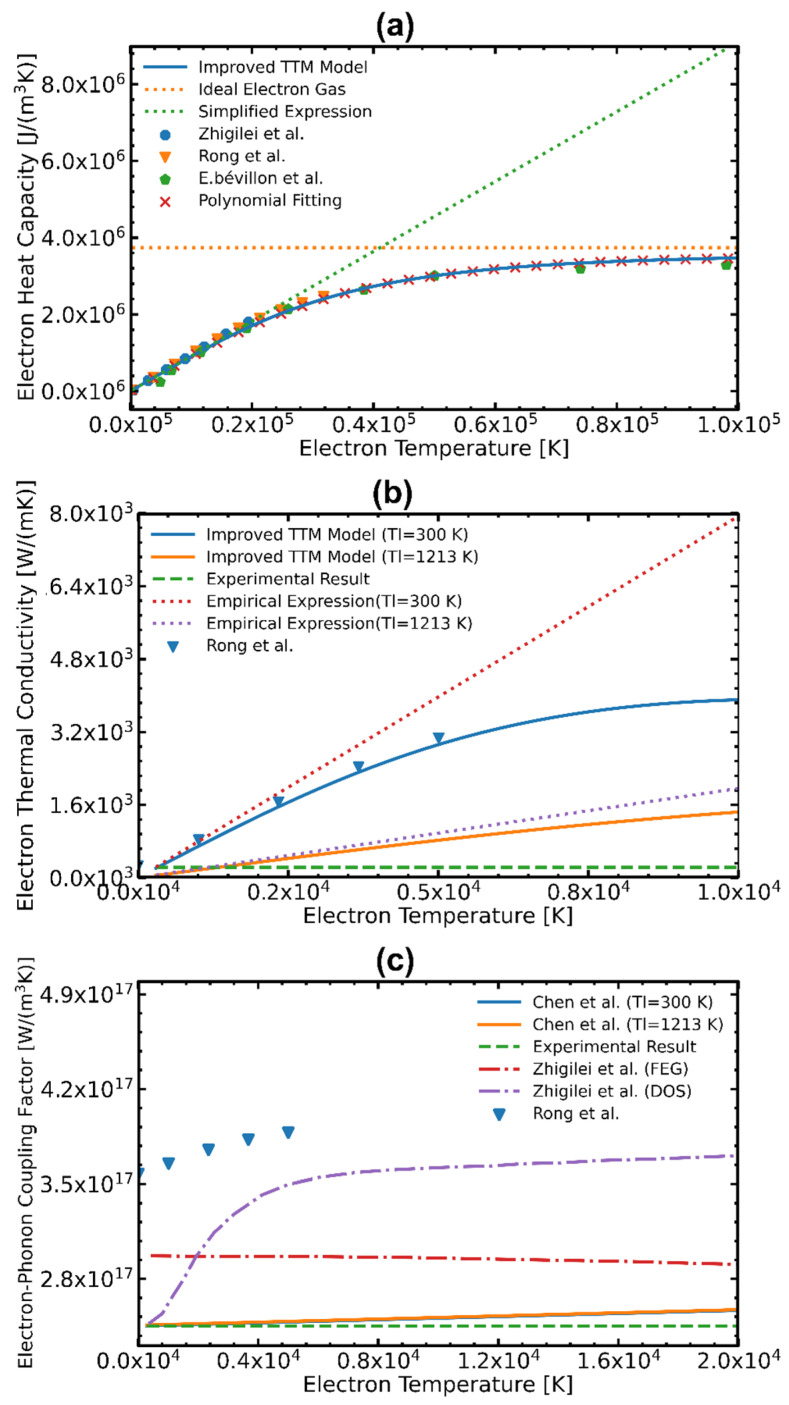
Temperature-dependent thermophysical parameters of Al. Comparison of (**a**) electron heat capacity $C_{e}$, (**b**) electron thermal conductivity $k_{e}$, and (**c**) the electron-phonon coupling factor $G_{e-ph}$ obtained by different calculation methods [17,46,54,55,56,57].

Figure 7 presents the thermophysical parameters of Al obtained through various methods. The solid, dashed, and dotted lines represent the results obtained from the full-run quantum treatment (Jiang et al. [54]), classical simplified expression (Ideal Electron Gas and Simplified Expression [55]), empirical formula (Chen et al. [46]), and ab initio QM (Zhigilei et al. [56], Rong et al. [17], and Bevillon et al. [57]), respectively. It is evident that $C_{e}$ is influenced by $T_{e}$ in Figure 7a. As $T_{e}$ increases, $C_{e}$ exhibits initial linear growth and then tends to approach a constant value. Figure 7b illustrates the different $k_{e}$. In contrast to the previous parameter, $k_{e}$ is affected by the lattice temperature, which decreases as the lattice temperature increases. Regarding $G_{e-ph}$, shown in Figure 7c, it is worth noting that the precise determination of $G_{e-ph}$ remains an unresolved issue. Based on the current findings, it can be confirmed that the $G_{e-ph}$ of Al exhibits an increasing trend with rising electron temperature.

In summary, in this study, the temperature-dependent $C_{e}$ is determined through the polynomial fitting. By taking $T_{l}$ into account with the empirical formula, the temperature-dependent $k_{e}$ and $G_{e-ph}$ are derived. The detailed expression is shown below:

1. For $C_{e}$

(8) $$\begin{array}{c} C_{e}=7.5209\times 10^{2}+8.8184\times 10^{1}T_{e}+5.5628\times 10^{-4}T_{e}^{2}-5.7927\times 10^{-8}T_{e}^{3}+9.7671\times 10^{-13}T_{e}^{4}\\ -7.1915\times 10^{-18}T_{e}^{5}-2.0170\times 10^{-23}T_{e}^{6} \end{array}$$

2. For *k_e_*

(9) $$k_{e}=\frac{1}{3}\nu_{f}^{2}\tau_{e}C_{e}=\frac{1}{3}\nu_{f}^{2}\frac{1}{\left(AT_{e}^{2}+BT_{l}^{2}\right)}C_{e}$$

3. For *G_e–ph_*

(10) $$G_{e-ph}=G_{0}\left[\frac{A}{B}\left(T_{e}+T_{l}\right)+1\right]$$

where $v_{f}$ is the fermi velocity. $A=1.39\times 10^{6}\;s^{-1}K^{-2}$ and $B=5.92\times 10^{11}\;s^{-1}K^{-1}$ are the material constants for Al [17]. $G_{0}=2.45\times 10^{17}(W/m^{3}K)$ is the initial electron-phonon coupling factor at room temperature [58].

**Figure 8:**
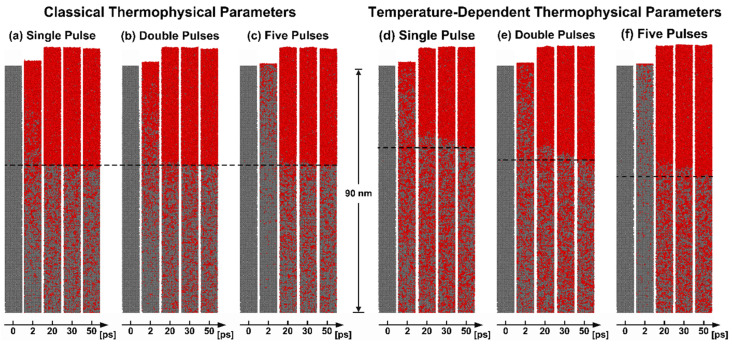
Atomic snapshots of the melting process on the Al surface region of 90 nm, the left is the classical thermophysical parameters group for (**a**) SP, (**b**) DP, and (**c**) FP, while the right is the temperature-dependent thermophysical parameters group for (**d**) SP, (**e**) DP, and (**f**) FP. The black dotted line represents the boundaries between the solid and liquid phases.

Figure 8 shows the melting process of the first 90 nm from the surface of Al after irradiation with different laser-pulse settings, with a total fluence of $350\;J/m^{2}$. Figure 8a–c represents the classical thermophysical parameters group, while Figure 8d–f corresponds to the temperature-dependent thermophysical parameters groups. During the 0−20 ps melting process in both groups, Al’s surface undergoes a relatively rapid melting stage. Subsequently, from 20 ps to 50 ps, the melting region expands at a velocity lower than the speed of sound, while a clear melting region boundary appears. This two-stage process exhibits similarities to the phenomenon observed in our previous work [43]. The appearance of obvious melting is delayed with the increase in the number of pulses. This delay is attributed to the longer thermal time of Al and the lower energy carried by individual laser pulses in the multi-pulse mode. Taking FP as an example, the total time required for the laser energy to be absorbed equals the sum of the total pulse intervals ($4\times t_{interval}$) and the time for a Gaussian beam ($6\times t_{p}$) to be fully absorbed, as shown in Figure 4—that is, 4.6 ps. Interestingly, the more obvious differences are evident in the visible liquid-phase region boundaries at 50 ps (black dot lines). In the case of the left group in Figure 8a–c, the impact of the change in pulse number is considered negligible. The variation in pulse numbers has minimal impact on the position of the melting region boundaries and the distribution of the liquidus point. However, for the temperature-dependent thermophysical parameter group in Figure 8d–f, more energy is transferred to a deeper position. Moreover, after the appearance of the boundary of the liquid phase region, there are still a large number of nucleation points below it, with liquid atoms comprising over 90% of the total number of atoms in some grids.

**Figure 9:**
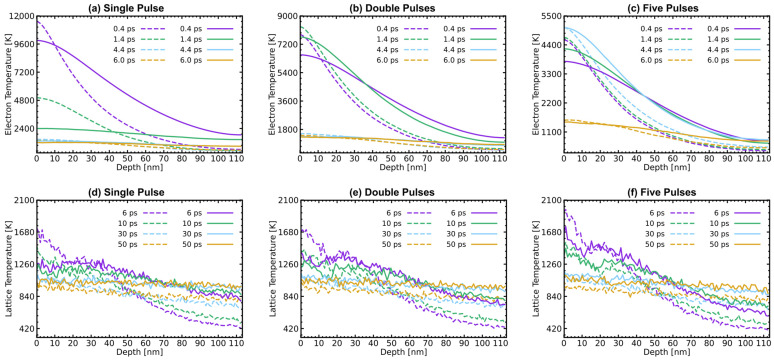
Spatial distributions of (**a**–**c**) electron temperature $T_{e}$, and (**d**–**f**) lattice temperature $T_{l}$ at different pulse numbers for (**a**,**d**) SP, (**b**,**e**) DP, and (**c**,**f**) FP. Dashed lines represent the classical thermophysical parameters group results and the solid lines represent the temperature-dependent thermophysical parameters group results.

The spatial distribution of electron and lattice temperatures at different time points is illustrated in Figure 9. In each sub-figure, the solid lines in the temperature-dependent parameter group exhibit a smoother lattice temperature change with increasing depth compared to the dashed lines in the classical parameter group. This distinction arises from the increased electron thermal conductivity and electron-phonon coupling factor at higher electron temperatures, resulting in enhanced energy transfer towards the deeper region. For electron temperature in Figure 9a–c, as the pulse number increases, the peak time of electron temperature is delayed (SP—0.5 ps, DP—0.8 ps, FP—4.6 ps), and the peak temperature of the surface is significantly reduced (classical parameters: SP—11,538.10 K, DP—8321.62 K, FP—5052.08 K, temperature-dependent parameters: SP—9905.54 K, DP—7665.19 ps, FP—5059.64 K). Regarding the lattice temperature in Figure 9d–f, an increment in pulse number leads to a higher lattice temperature. It is worth noting that the application of temperature-dependent parameters will increase the lattice temperature at a depth greater than 30 nm, which is consistent with previous results [17,43]. This phenomenon is also the reason for the observed enlargement of the melting region shown in Figure 8.

In conclusion, the introduction of temperature-dependent thermophysical parameters can effectively reflect the phenomenon that multiple pulses increase the melting depth (first 50 ps). The increase in pulse number can significantly reduce the surface electron temperature. This causes the lattice sub-system to obtain more energy, thus increasing the lattice temperature. It is worth emphasizing that the enhancement effect of multiple pulses only occurs when the pulse energy is far below the ablation threshold ($640\;J/m^{2}$). When the pulse energy approaches the ablation threshold, the melting depth will be affected by the material size, surface expansion, and ablation phenomena and shows no increase or even a decrease [13,59]. Furthermore, the mentioned enhancement effect only occurs in the early stage. Nevertheless, the current results are sufficient to demonstrate that the MD-TTM model incorporating temperature-dependent thermophysical parameters can effectively achieve the control mechanism of Al’s thermophysical properties by pulse train.

### 3.3. The Influence of Multi-Pulse on the Phenomenon of Lateral Melting

**Figure 10:**
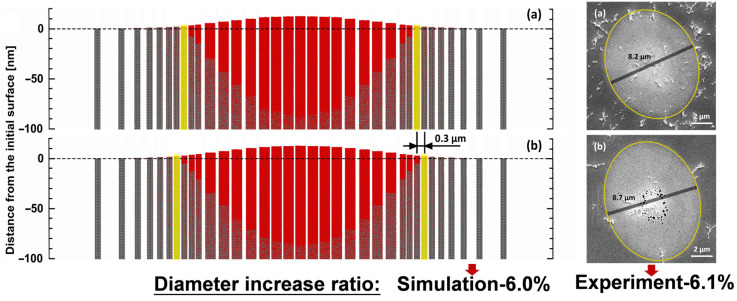
The morphology of the 2D melting region (50 ps) of the Al surface at (**a**) SP and (**b**) DP with a 2 ps interval, the left is the simulation results, and the right is the experimental results.

Figure 10a,b depicts the morphology of the 2D melting region of the Al surface at 50 ps, under the same laser energy, for both the SP and DP with a 2 ps interval, respectively. Among them, the left is the simulation results, and the right is the experimental results. To facilitate a direct comparison between the simulated results and the experimental results presented in Section 3.1, the laser energy at the center of the simulation is set to $440\;J/m^{2}$, which is below the ablation threshold ($640\;J/m^{2}$ [37,38]).

In the results of the SP, it is observed that melting occurs within ~5.1 μm of the farthest position from the center of the laser spot, and the fluence at the position is $160\;J/m^{2}$, representing the melting threshold for the SP. In the results of the DPs, this position expands to ~5.4 μm, with a fluence of $140\;J/m^{2}$. Combining the results from Section 3.2, it is found that DPs can reduce the melting threshold compared to the SP, thereby enlarging the diameter of the melting region.

According to the above results, we speculate the cause of the void. During the subsequent development of the melting process, the atoms with more kinetic energy in the central region flow toward the lower liquid level in the edge region. As the liquid melting region resolidifies into a solid state, the surface expansion decreases, resulting in the formation of irregular voids. The larger diameters of the melting region observed for the DP indicate a higher kinetic energy of Al at the surface during liquefaction. This phenomenon may be the cause of the generation of irregular voids near the center for a DP with a 2 ps interval, as discussed in Section 3.1. Combining this with the conclusion in Section 3.4 (that the early-stage melting depth is larger for a DP with a 2 ps interval compared to a 3 ps interval), the phenomenon of a more concentrated distribution of voids in the SEM image of the DP with a 3 ps interval in Figure 6d may be caused by the lower kinetic energy of liquid atoms in the melting region.

**Figure 11:**
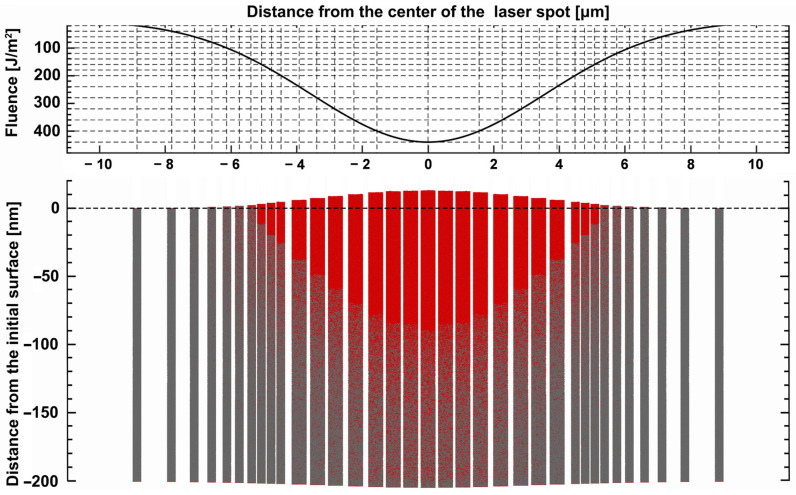
The morphology of the 2D melting region (50 ps) of the Al surface at FP with a 2 ps interval.

Figure 11 presents the predicted results for FP under the same conditions discussed in this section. The results of FP exhibit larger melting diameters and depths, consistent with the previous discussions (Section 3.2).

Such results mean that it is possible to achieve an adjustment of the melting phenomenon through reasonable pulse design (pulse number, total pulse energy, pulse interval, and pulse energy ratio).

### 3.4. The Influence of Pulse Interval and Pulse Energy Ratio on the Longitudinal Melting Phenomenon

**Figure 12:**
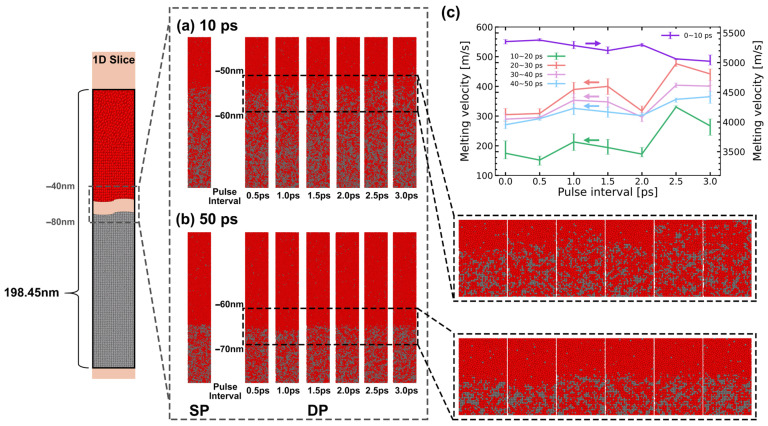
The longitudinal evolution of the melting region during the first 50 ps under both SP and DPs with varying pulse intervals. (**a**) atomic snapshots at 10 ps, (**b**) atomic snapshots at 50 ps, and (**c**) melting speed in different stages.

Due to the lack of experimental observation methods for longitudinal melting phenomena, this section exclusively relies on simulation to predict and analyze the results of longitudinal ultrafast melting. Figure 12 illustrates the longitudinal evolution of the melting region during the first 50 ps, irradiated by both the SP and DPs with varying pulse intervals. Consistent with the description in Section 3.2, Al mainly undergoes a rapid melting stage dominated by homogeneous melting and a slow melting stage dominated by heterogeneous melting [43]. MD snapshots at 10 ps in Figure 12a and 50 ps in Figure 12b are presented to describe the evolution of melting depth at different melting stages. Furthermore, the average velocities for the five stages are plotted in Figure 12c.

During the initial 0−10 ps, the melting velocities for all pulse intervals and pulse numbers are significantly higher compared to subsequent stages, necessitating the use of the right *y*-axis for distinction, as shown in Figure 12c. At 10 ps, there is little difference in melting depth between the SP and DPs, whereas the influence of pulse intervals becomes more pronounced. The maximum melting velocity is observed at the DP with a 0.5 ps interval (5379.6 m/s). As the pulse interval increases, there is an overall decreasing trend in the average velocity during the rapid melting stage. Surprisingly, at a 2 ps pulse interval, the melting velocity is higher than at a 1.5 ps pulse interval. As the melting process further evolves, between 10 ps and 40 ps, the melting velocities decrease to below the speed of sound. The depth differences resulting from pulse intervals gradually decrease.

**Figure 13:**
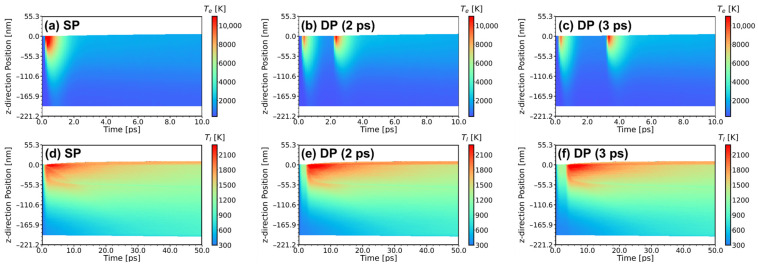
Evolution of electron temperature $T_{e}$ and lattice temperature $T_{l}$ under different pulse settings (**a**,**d**) SP, (**b**,**e**) DP with a 2 ps interval, (**c**,**f**) DP with a 3 ps interval.

To provide a more detailed analysis of the mechanisms underlying the variations in melting velocity, Figure 13 shows contour plots of the evolution of electron temperature and lattice temperature with different pulse settings: SP, DP with a 2 ps interval, and DP with a 3 ps interval.

For the SP, the electron temperature at the Al surface easily reaches peak value (13,914.11 K). However, due to the faster electron heat conduction compared to the lattice heat conduction, thermal rapidly diffuses from the surface “hot” electron region to the deep “cold” electron region, reducing the electron temperature, as seen in Figure 13a. Consequently, the thermalization time of the lattice is limited, leading to a smaller range of high lattice temperature regions, indicated by the red color in Figure 13d.

For the DP with a 2 ps interval, although the peak electron temperature generated by the first laser pulse decreases compared to the SP (10,811.35 K), due to the irradiation from the second laser pulse, the electron keeps a high temperature during the initial 4 ps, as shown in Figure 13b. This increases the lattice thermalization time. The lattice sub-system gains more energy, forming a larger range of high-temperature regions, indicated by the red and orange colors in Figure 13e.

For the DP with a 3 ps interval, the increasing pulse interval between the two sub-pulses allows for sufficient relaxation between the hot electron sub-system and the cold lattice sub-system. When the time the second sub-pulse arrives, the electron temperature reduces to 1212.68 K, and the thermophysical parameter changes caused by the first sub-pulse have less effect on the absorption of the second sub-pulse. In other words, as the pulse interval continues to increase, the melting phenomena of the two sub-pulses become more independent, leading to a decreasing trend in the surface melting phenomenon.

Furthermore, as indicated in Section 3.2, electron thermal conductivity and the electron-phonon coupling factor are positively correlated with electron temperature. Combining the conclusions of [60], the effects of electron thermal conductivity and electron-phonon coupling factor are contradictory on lattice heating. The increase in electron thermal conductivity weakens the lattice heating, and a smaller melting depth is obtained. At the same time, the increase in the electron-phonon coupling factor enhances the lattice heating, and a larger melting depth is obtained. The peak velocity at 2 ps implies that the influence of changes in the electron-phonon coupling factor on lattice thermalization is greater than that of electron thermal conductivity. This facilitates the energy absorption of the lattice sub-system from the second sub-pulse, resulting in a greater melting velocity and melting depth. However, when the pulse interval is greater than 2 ps, during the irradiation of the second pulse, both the electron and lattice have sufficient time to relax and conduct heat deeper into the Al, leading to a “cooler” electron temperature at the surface. Consequently, the melting velocity decreases again, as shown in Figure 13c,f.

**Figure 14:**
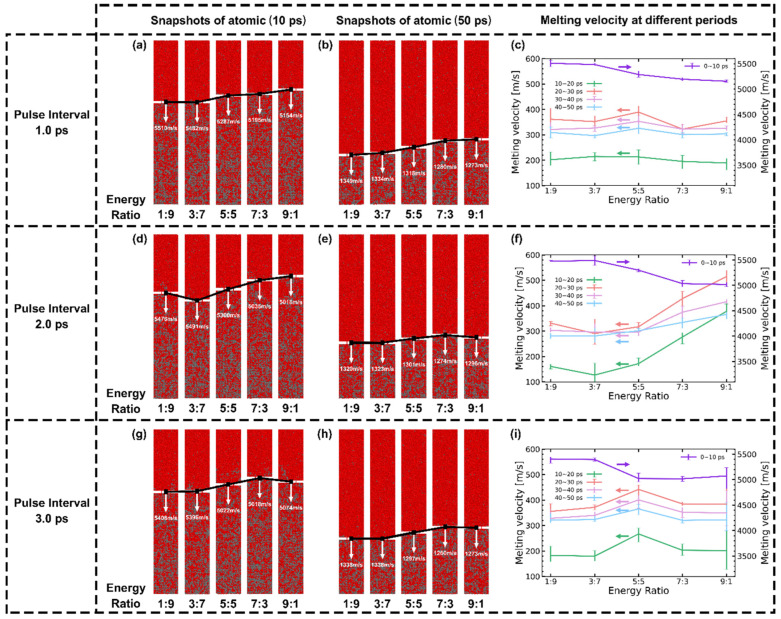
The impact of sub-pulses energy ratio of DP on the melting process. Sub-figures for DPs with (**a**–**c**) 1 ps, (**d**–**f**) 2 ps, and (**g**–**i**) 3 ps intervals, respectively. The first and second columns are atomic snapshots of 0−10 ps and 0−50 ps. The third column represents the melting speed vs. energy ratio at different stages.

Figure 14 illustrates the impact of the pulse energy ratio of sub-pulses for DPs on the melting process. During the initial 10 ps of the rapid melting stage, compared to the 1:1 and decreasing energy train, an increasing energy train results in a greater amplification of melting velocities, particularly evident at a 2 ps interval with a 3:7 energy ratio configuration.

The simulation results in this section can be summarized as follows: (i) For the rapid melting stage (0−10 ps), the melting depth is influenced by the pulse interval and pulse energy ratio. A DP with a 2 ps interval and a pulse energy ratio of 3:7 exhibits the maximum melting depth. (ii) For the whole process of melting, it is observed that as the simulation time increases, the differences in melting depth gradually decrease. Predictably, due to the consistent total input energy, the final melting depth is independent of the pulse interval and energy ratio. This corresponds to the results presented in [13,59,61].

## 4. Conclusions

This study uses the femtosecond laser-pulse train melting experiment and the molecular dynamics coupling two-temperature model (MD-TTM model) incorporating temperature-dependent thermophysical parameters to investigate ultrafast melting processes of Al irradiated by different laser-pulse settings. The investigation encompasses variations in pulse number, pulse interval, and pulse energy ratio to explore both lateral and longitudinal melting processes. The research findings indicate that: (i) the MD-TTM model with temperature-dependent property parameters can effectively reflect the changes in Al’s thermophysical properties irradiated by the femtosecond laser train. This model provides theoretical support for the results of femtosecond laser multi-pulse experiments. (ii) As the pulse number increases, the melting threshold at the edge region of the laser spot decreases, resulting in an increase in the melting region diameter. (iii) At the same laser energy (much less than the ablation threshold), the melting depth of Al in the early stage increases with the increase in pulse number. Specifically, melting depth and melting velocity in the early stage reach the maximum for a DP with an increasing energy ratio (3:7) and a 2 ps pulse interval. The pulse number, pulse interval, and energy ratio only influence melting depth and melting velocity in the early stage. This confirms that the independent control of melting phenomena can be achieved through pulse train. This will contribute to the improvement of processing quality in additive and subtractive manufacturing applications using femtosecond laser technology.

## Figures and Tables

**Table 1:** The lattice temperature obtained by the simulation results with different thicknesses and pulse numbers.

	Pulse Number	1	2	3	4	5	Standard Deviation, σ	
Thickness [nm]								
50.7		1296.4	1284.9	1286.3	1281.8	1286.9	4.9	
113.4		1014.8	1010.9	1015.0	1016.2	1011.9	2.0	
198.45		795.5	784.4	780.9	783.7	777.8	6.0	
396.9		641.6	629.7	627.4	627.8	626.7	5.6	
510.3		505.6	501.7	499.0	495.4	495.8	3.8	

## Data Availability

Please contact the corresponding author for data related to this article.

## Nomenclature

A	Material constant for Al, $s^{-1}K^{-2}$
B	Material constant for Al, $s^{-1}K^{-1}$
$C_{e}$	Electron heat capacity, $J/(m^{3}K)$
$C_{l}$	Lattice heat capacity, $J/(m^{3}K)$
$F_{ab}$	Absorb laser fluence, $J/m^{2}$
$F_{i}$	Total force exerted on atom i, N
$F_{o_{\Delta }}$	Fourier number, none
${\tilde{F}}_{ran}$	Random force, N
$G_{0}$	Initial electron-phonon coupling factor, $W/(m^{3}K)$
$G_{e-ph}$	Electron-phonon coupling factor, $W/(m^{3}K)$
i	Pulse number of the current calculation, none
$K_{e}$	Total kinetic energy in the grid, J
$k_{B}$	Boltzmann constant, J/K
$k_{e}$	Electron thermal conductivity, W/(mK)
$k_{l}$	Lattice thermal conductivity, W/(mK)
$L_{ball}$	Ballistic transportation length, m
$L_{op}$	Optical penetration depth, m
$m_{i}$	Mass of atom i, kg
N	Atom density, $1/m^{3}$
$N_{laser}$	Total pulse number, none
R	Reflectivity, none
S	Laser source term, $J/m^{3}$
$T_{e}$	Electron temperature, K
$T_{l}$	Lattice temperature, K
t	Time of the current calculation, s
$t_{i}$	Pulse interval between the *i*th and (*i* − 1)th sub-pulses, s
$t_{p}$	Laser-pulse duration, s
$v_{i}$	Velocity of atom i, m/s
${\tilde{v}}_{i}$	Random vector, m/s
z	Depth of the laser irradiation, m

**Greek symbols**

α	Thermal diffusivity, $m^{2}/s$
$\gamma_{i}$	Friction term of atom i, kg/s
Δt	Timestep, s
Δx, Δy, Δz	Spacesteps, m
$\eta_{i}$	Proportion of the ith pulse to the total energy, none
σ	Standard deviation, none
$\tau_{e}$	Electron relaxation time, s
